# Developing and characterizing a single-domain antibody (nanobody) against human cytotoxic T-lymphocyte-associated protein 4 (hCTLA-4)

**DOI:** 10.22038/IJBMS.2021.57774.12851

**Published:** 2021-09

**Authors:** Nazli Sotoudeh, Zahra Noormohammadi, Mahdi Habibi-Anbouhi, Fatemeh Kazemi-Lomedasht, Mahdi Behdani

**Affiliations:** 1 Department of Biology, Science and Research Branch, Islamic Azad University, Tehran, Iran; 2 National Cell Bank of Iran, Pasteur Institute of Iran, Tehran, Iran; 3 Biotechnology Research Center, Venom and Biotherapeutics Molecules Laboratory, Pasteur Institute of Iran, Tehran, Iran; 4 Zoonoses Research Center, Pasteur Institute of Iran, Amol, Iran

**Keywords:** CTLA-4 antigen, Immune checkpoint- proteins, Immunotherapy, Nanobody, Single-domain antibodies

## Abstract

**Objective(s):**

Cytotoxic T-lymphocyte-associated protein-4 (CTLA-4) is the most important human immune checkpoint that modulates T cells activity and brings about immune-homeostasis. Accordingly, checkpoint inhibitor cancer therapy has been approved as a growing method to block over-expressed immune checkpoints, such as CTLA-4 receptors. Considering the competitive characteristics of single-domain antibodies with monoclonal antibodies, we tried to develop a camelid Nanobody against human CTLA-4.

**Materials and Methods:**

We have constructed the VHH gene library by using immunized-camel peripheral blood mononuclear cells and carrying out the Nested-PCR technique. VHH-library was screened by phage display technique and specific nanobodies against CTLA-4 protein were selected and amplified with bio-panning steps. Stronger binders were screened by Periplasmic Extract-ELISA, followed by estimating the complexity of the library. Specific anti-CTLA-4 Nanobody and 3hCTL55, with longer CDR3 and a higher binding rate, were selected for more assays.

**Results:**

Results revealed the existence of two different clones in the library with 10^8^ binders. In comparison with seven different antigens, using the ELISA technique confirmed the specificity of Nanobody 3hCTL55 against human CTLA-4 antigen. We calculated Nanobody 3hCTL55 affinity for human CTLA-4 antigen at 50×10^-9^ M, approximately. Performing western blot and Flow-cytometry techniques showed that Nanobody 3hCTL55 was able to specifically detect and attach both commercial human CTLA-4 protein and human CTLA-4 antigen on the cell surface and in the cell lysate.

**Conclusion:**

Taken together, this developed camelid-specific anti-CTLA-4 Nanobody 3hCTL55, selected from a high-quality immune library by phage display technique, may be effective for further study about cancer diagnosis and cancer-therapy purposes.

## Introduction

Cytotoxic T-lymphocyte-associated protein 4 (CTLA-4), known as a cluster of differentiation CD152, is a key human immune checkpoint molecule. CTLA-4 is a member of the immunoglobulin superfamily that acts as a co-inhibitory receptor and shares the same ligands, B7s, with the co-stimulatory receptor, CD28. After T cell activation, CTLA-4 receptors predominantly express on activated T lymphocytes, competitively bind to b7-ligands, and induce inhibitory signals through both cell-extrinsic and intrinsic mechanisms (1, 2). This interaction eventually leads to a decrease in T cell proliferation and differentiation, cell cycle progression, and cytokine production. Consequently, CTLA-4 down-regulates immune responses and brings immune homeostasis (3, 4).

Significantly, over-expression of checkpoint inhibitory molecules, such as CTLA-4, was presented as a strategy of cancer cells to evade the immune system. This may indicate the important role of CTLA-4 action in regulating anti-tumor responses (5, 6). Different studies revealed the existence of an association between CTLA-4 gene polymorphisms and numerous cancers, for instance; melanoma, breast cancer, non-small cell lung cancer (NSCLC), skin cancer, gastric cancer, colorectal cancer, and many others (7). Although the CTLA-4 expression profile does not stay steady along with cancer progression, the over-expression of CTLA-4 seems to be a noticeable cancer-biomarker (8, 9). In this regard, clinical achievements in cancer therapy with checkpoint inhibitors (CPI) revealed blocking inhibitory immune checkpoint molecules such as CTLA-4 and human programmed cell death protein 1 (PD1) resulted in significant therapeutic approaches (10). Noticeably, anti-CTLA-4 molecules enable the anti-tumor activity of T cells and reduce inhibitory signals through different mechanisms (11). FDA has approved several monoclonal antibodies (mAbs) as CPIs for different cancer types (12). Heavy-chain Antibodies (HcAbs) are another group of immunoglobulins with a molecular weight of ~90 kDa. HcAbs only consist of two heavy chains and have no CH1 domain (13). The antigen recognition site of HcAbs is composed of a single variable domain, called VHH or Nanobody (Nb). Due to the distinct advantages of nanobodies, in comparison with conventional mAbs, they are proposed to be more potential diagnostic and therapeutic agents for cancer immunotherapy (14). Nbs are very small, (2.5×4 nm/~15 kDa), which enables them to achieve greater microenvironment penetration (15). These molecules, with an almost conserved sequence, have a “Lego-like” structure and are easily manipulated to combine other molecules for different purposes (16). In comparison with Abs, Nbs are more soluble and more stable in harsh conditions such as extreme temperature and pH. Because of having high-order homology with the human VH domain, Nbs are less immunogenic for humans. Adding to all mentioned beneficial properties, low costing production is another reason that all together encourage scientists to focus on developing Nbs for a variety of research (15).

This study aimed to construct a novel camelid immune library against human recombinant CTLA-4 antigen and produce the strongest and the most specific anti-CTLA-4 Nanobody which was followed by Nanobody characterization with different techniques (17-19). 

## Materials and Methods

***Camel immunization and lymphocyte separation***

Nine-month-old female *Camelus dromedarius* was immunized with 100 µg of recombinant human extracellular domain of CTLA-4 protein (17), mixed with an equal volume of Freund’s complete adjuvant for the first time, and Freund’s incomplete for subsequent injections. After six subcutaneous injections with one-week intervals, an upward trend of the immune response was approved by the serum-ELISA test (17). Whole blood samples were collected as a Nanobody-gene pool, from the immunized camel. Blood was diluted with an equal volume of phosphate-buffered saline (PBS) and then Peripheral Blood Mononuclear Cells (PMBCs) were isolated by density gradient centrifugation at 400 × g for 30 min with Ficoll solution. Isolated PMBC was divided into 5×10^7^ aliquots by using freeze medium culture (90% fetal bovine serum and 10% dimethyl sulfoxide), and then stored at -70 ^°^C for the next step.

***VHH cloning and library construction***

Total RNA was obtained from 5×10^7^ PMBC with RNeasy Mini Kit (Qiagen, Germany), and 40 µg of RNA was used for cDNA synthesizing. Following that, we carried out Nested PCR to amplify VHH gene fragments. Briefly, cDNA was undergone the first run of PCR with the first set of primers; CALL001 (leader sequence-specific primer) GTC CTG GCT GCT CTT CTA CAA GG, and CALL002 (CH2-specific primer) GGT ACG TGC TGT TGA ACT GTT CC. Next, 700 bp amplicons were purified from 2% agarose gel and used as a template for the nested primers; VHH-A6E (Framework 1-specific primer) GAT GTG CAG CTG CAG GAG TCT GGR GGA GG) and VHH-38 (Framework 4-specific primer) GGA CTA GTG CGG CCG CTG GAG ACG GTG ACC TGG GT, introducing *Pst*I and *Not*I restriction site sequences, respectively. Moreover, the first PCR was set up with annealing temperature at 47.8 ^°^C for 30 sec and 30 amplification cycles which changed to 55 ^°^C for 30 sec and 20 amplification cycles for the second PCR. The second PCR fragments were then cleaned and digested with *Pst*I and *Not*I enzymes for 16 hr at 37 ^°^C. Next, the digested fragment was ligated into the pHEN4 vector, containing Hemagglutinin (HA)-tag at C-terminal, with incubation overnight at 16 ^°^C. The purified ligated mixture was transformed into electrocompetent *Escherichia* *coli* TG1 cells and after one-hour incubation at 37 ^°^C, cells were cultured on Luria-Bertani (LB) agar plates, containing 100 µl ml-1 ampicillin and 2% (wt/vol) glucose. To calculate the electroporation efficiency and library size, 100 µl of four tenfold serial dilutions were plated on separate LB agar plates. In addition, we carried out colony-PCR for some random colonies with GIII (CCA CAG ACA GCC CTC ATA G) and RP (TCA CAC AGG AAA CAG CTA TGA C) primers to measure the insertion rate.

***CTLA-4 specific phage selection***

The bacterial library was converted to the phage VHH library by applying the helper phage VCSM13 (Stratagene, La Jolla, CA, USA). Then, precipitated phages were used for 3 successive bio-panning rounds. Flat-bottom 96-well immunoplates (Nunc maxisorp, Roskilde, Denmark) were coated with 5 µg hCTLA4 in 100 µl carbonate-bicarbonate buffer (pH 9.2) and immobilized at 4 ^°^C, overnight. The negative control well was incubated with 100 µl carbonate-bicarbonate buffer. Wells were blocked by 4% skim milk for one hour at Room temperature (RT). After twice washings with PBS, 100 µl of the phage library (10^11^ particles) were added to wells and incubated for one hour at RT. To eliminate non-bounded phage particles, wells were washed with PBS-0.05% Tween-20 (PBST) 10 times, which increased to 20 x and 25 x for second and third panning rounds, respectively. High selective bound phages were eluted with triethylamine solution (100 mM, pH 10.0) for 10 min and then immediately was neutralized with Tris–HCl (1 M, pH 8.0). To amplify phages for the next round of selection, *E. coli* TG1 cells were infected by eluted phages, followed by adding 10^11^ particles of VCSM13 helper phages, for phage rescuing. After three rounds, polyclonal phage ELISA was performed on the hCTLA-4 antigen, to evaluate the enrichment rate of specific phages (18). We picked sixty-four random single colonies to screen with the periplasmic extract ELISA (PE-ELISA) method. Briefly, colonies were cultured and protein expression was induced with 1M IPTG (overnight (O/N) at 28 ^°^C). Then periplasmic proteins were extracted through sucrose osmotic shock and used for the PE-ELISA (19). A plasmid of positive single colonies was extracted with a plasmid extraction kit (Vivantis, Malaysia) and screened by DNA sequencing (Gene Fanavaran Co.). Further, sequencing results were analyzed in National Center for Biotechnology Information-Basic Local Alignment Search Tool (NCBI-BLAST).

***Large scale expression of VHH and purification***

For protein expression, we selected one colony entitled Nb 3hCTL55 for more characterization. An encoding 55 Nb construct was amplified by PCR and digested with *Pst*I and *BstE*II and subcloned in the pHEN6-C vector. The new recombinant vector was introduced into competent *E. coli WK6 *with the heat-shock method. Subsequently, a transformed single colony was cultured in 1L LB broth (100 μg ml^-1^ ampicillin) and induced by 1 mM Isopropyl β- d-1-thiogalactopyranoside (IPTG) (28 °C, 18 hr). Cells were pelleted by centrifuge (3000 × g for 15 min), followed by objecting osmotic shock to achieve periplasmic extract containing the VHH-His tag. Nb 3hCTL55 purification was done by His-select Ni-NTA affinity resin (Thermo Fisher Scientific, USA) and then was eluted with PBS-0.5 M imidazole. Elution was concentrated on an Amicon Ultra-2 ml Centrifugal Filter (Merck Millipore, Darmstadt, Germany) with a molecular mass cutoff of 10 kDa. For more confirmation, protein concentration was determined at OD_280_ nm and the quality of purification was evaluated by 15% SDS-PAGE and western blotting. Briefly, after SDS-PAGE, bands were transferred from gel to nitrocellulose membrane. Anti-camel antibodies were used to detect Nb. Visualizing was done by 3,3’-diaminobenzidine (DAB). 

***Nanobody characterization***

***Antigen binding specificity***

We estimated antigen-binding specificity of Nb 3hCTL55 on numerous proteins; human PD1 (Genscript Biotech, USA), recombinant mouse PD1, human programmed death-ligand 1 (hPDL1), (Genscript Biotech, USA), recombinant mouse PDL1, human CTLA-4 (Genscript Biotech, USA), recombinant mouse CTLA-4, casein (Sigma-Aldrich, USA), and bovine serum albumin (Sigma-Aldrich, USA). All washing steps were done three times with 300 µl PBS 1x. ELISA plate was coated with 1 µg/mL of proteins. After blocking with 4% skim milk, wells were washed and incubated with 100 μl of the anti-hCTLA-4 Nb (1 μg/ml) for 2 hr at 37 ^°^C. Then, with washing intervals, 100 μl of the anti-HA tag antibody (1/2000), followed by 100 μl of anti-mouse Horse Radish Peroxidase anti-mouse Horse Radish Peroxidase (HRP) conjugate antibody (1/2000) were added to wells for 1 hr at RT, respectively. After the final washing steps, positive wells were screened by adding 100 μl of 3,3′,5,5′-tetramethylbenzidine (TMB) (Sigma, Germany) solution. The enzymatic reaction was stopped by 2N sulfuric acid and results were considered by measuring the absorbance at 450 nm.

*Affinity*

The affinity of Nb 3hCTL55 against the CTLA-4 antigen was determined by the Beatty ELISA technique (20). Briefly, the CTLA-4 antigen was diluted with carbonate–bicarbonate buffer (pH 9.2) in two different concentrations (10 & 1 µg/ml) and coated in a 96-well plate at 4 ^°^C, overnight. Bovine-Serum-Albumin (BSA) with the same concentration was used as a negative control. All were set up in triplicate. After blocking with 4% skimmed milk, an increasing series (0-100 nM) of Nb 3hCTL55 concentration was added to each well. Then, the ELISA protocol was carried out as described before. Finally, absorbance at 450 nm was measured and the K_aff_ value was calculated based on the modified Beatty method (21).

*Western blotting on cell lysate*

Reactivity of Nb 3hCTL55 with CTLA-4 antigen was assessed with the western blot method. We used commercial human CTLA-4 protein (CTLA-4 Fc Chimera, Human, Genscript, USA) and CTLA-4 protein from cell lysate for this examination. Briefly, Jurkat cells (CTLA-4 positive cell line) and HEK-293 (CTLA-4 negative cell lines) were cultured in RPMI-10%FBS and DMEM-10%FBS medium, respectively. Both cell lines were obtained from the National Cell Bank of Pasture Institute of Iran. Next, 1×10^6^ cells were harvested and washed twice with PBS and then re-suspended in a non-reduced lysis buffer. Cell lysate and commercial human CTLA-4 protein were exposed on 15% SDS-PAGE gel and proteins were blotted on nitrocellulose paper. After overnight blocking with 4% skim milk, the membrane was incubated (for one hour at RT) with Nb 3hCTL55, Rabbit anti-His (1/2000), and anti-Rabbit HRP-conjugate, respectively. Interval washings were applied three times with PBST. Finally, bands were visualized with DAB. 

*Flow cytometry*

We evaluated the binding potency of the selected Nb 3hCTL55 to the native CTLA-4 receptor expressed on the cell surface of the Jurkat cell line by flowcytometry. Briefly, Jurkat cells (CTLA-4 presenting cells) were washed 2 times with cold PBS-2% FBS and aliquot up to 1x10^6^ cells per ml. Next, cells were incubated with 2 µg of purified Nb 3hCTL55 for 1 hr on ice and then with 1 µg of mouse anti-His for 1 hr. To monitor the conjugated molecules, we used 1 µg of anti-mouse Phycoerythrin (PE) conjugate. Finally, cells were washed and fluorescent intensity was measured by using the FL1 channel of the flowcytometer (Cyflow, Partec, Germany). Commercial anti-hCTLA-4 (BioLegend, USA), was used for positive and negative control, respectively. Data analysis was done via FlowJo software v. 7.6 (Tree Star, USA). 

***Statistical analysis***

All experiments were repeated twice, in triplicate wells. In statistical analysis, values of *P*<0.05 were considered significant, which were shown by using a two-tailed unpaired t-test. Regression performing and curve fittings were done with GraphPad Prism 7 (GraphPad Software, Inc., La Jolla, CA, USA). 

## Results

***Immunization and nanobody library construction***

**Figure 1:**
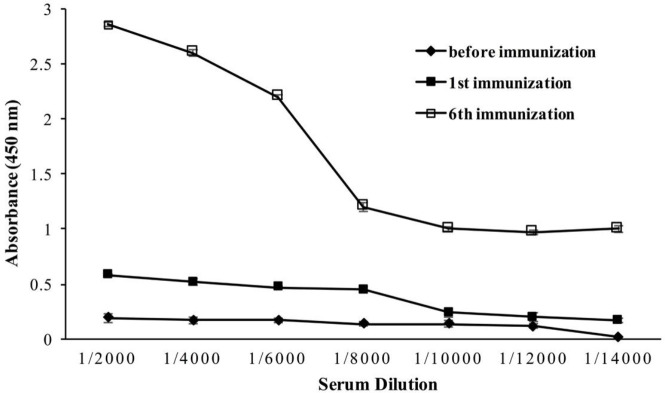
Camel immunization. Camel immune response against recombinant human CTLA-4 antigen was boosted after six subcutaneous injections

Applying six scheduled injections resulted in boosting the humoral immune response against recombinant human CTLA-4 protein, as reported previously (17). The ELISA was done with a diluted immunized camel sera sample. Total IgG titers marked up to 1/14000 and showed an upward trend of camel immunization after each boost injection (Figure 1). 

**Figure 2:**
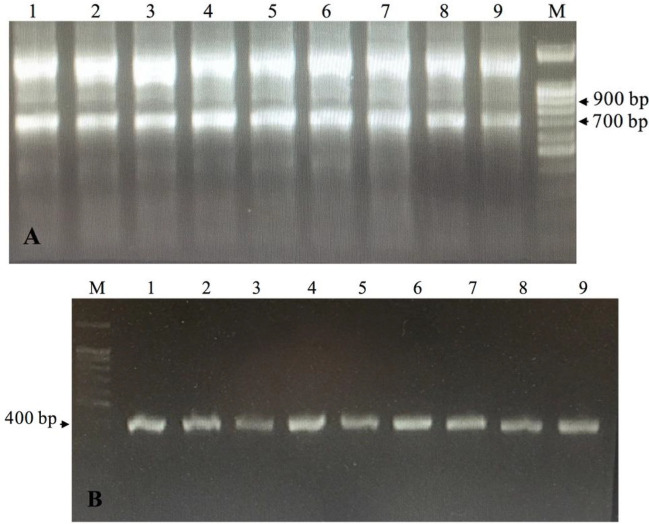
Extracted RNA from Camel lymphocytes was turned to cDNA and used for amplifying VHH genes by Nested-PCR. A) PCR-1. Using the first pair of primers, Call001 and Call002, resulted in 900 and 700 bp bands. 1-9; Nine different PCR reactions on 2% agarose gel M; 100 bp DNA ladder (Sinaclon). B) PCR-2. Using the second pair of primers, A6E and 38, amplified the VHH gene (400 bp). 1-9; Nine different PCR reactions on 1% agarose gel. M; 100 bp DNA ladder (Sinaclon)

**Figure 3:**
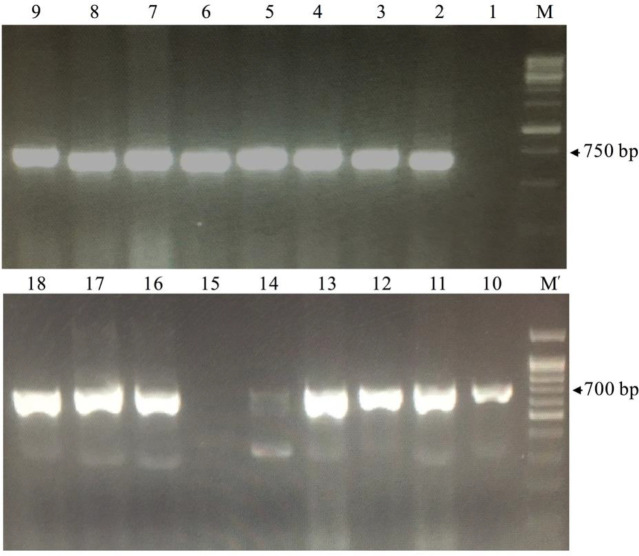
Transformation efficiency. pHEN4 plasmid containing the VHH gene was transformed to electro-competent *Escherichia coli* TG1 cells and the efficiency of transformation was estimated by random colony PCR with GIII and RP primers that resulted in 700 bp bands. Lane 1; Negative control, Lanes 2-18; Results of 17 random PCR colonies, M; 100 bp DNA ladder (Sinaclon). M; 1kb DNA ladder (Sinaclon)

To construct the library, the RNA of PMBC was used as a template in PCR. Consequently, the first PCR generated two distinct PCR amplicons with 900 bp (VH), and also 700 bp (originates from HcAb-encoding mRNA/VHH)(Figure 2A). The second PCR amplified only VHH sequences with 400 bp (Figure 2B). Following transformation, appeared colonies were counted from the gradient diluted plates and revealed that the library had approximately 10^8^ independent transformed bacteria. Transformation efficiency was estimated at 90% with the colony PCR method (Figure 3). Colonies were collected, mixed with 1% glycerol, and stored at -70 ^°^C.

***Panning and nanobody selection***

**Figure 4:**
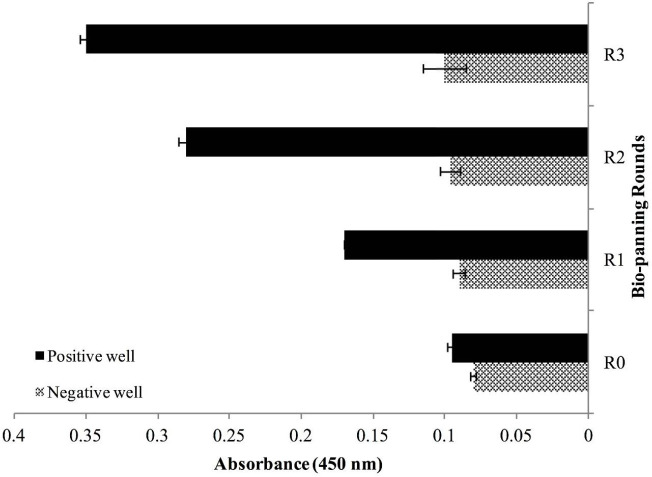
Phage-ELISA. Comparing phages gathered from three successive bio-panning rounds shows upward-trend enrichment

**Figure 5:**
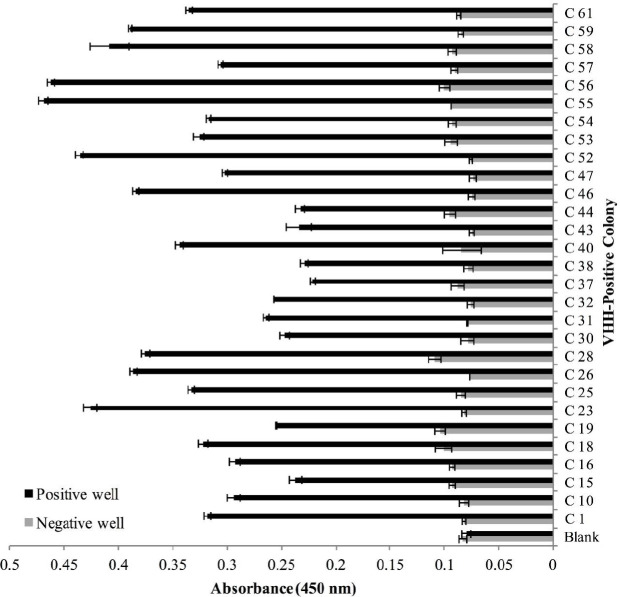
PE ELISA; screening single colonies expressing VHH with highest potent to bind to coated hCTLA-4 using ELISA technique. C x; (C) indicates colony and (x) indicates the number of colonies, colonies with greater than 0.2 optical density value at 450 nm

**Figure 6:**
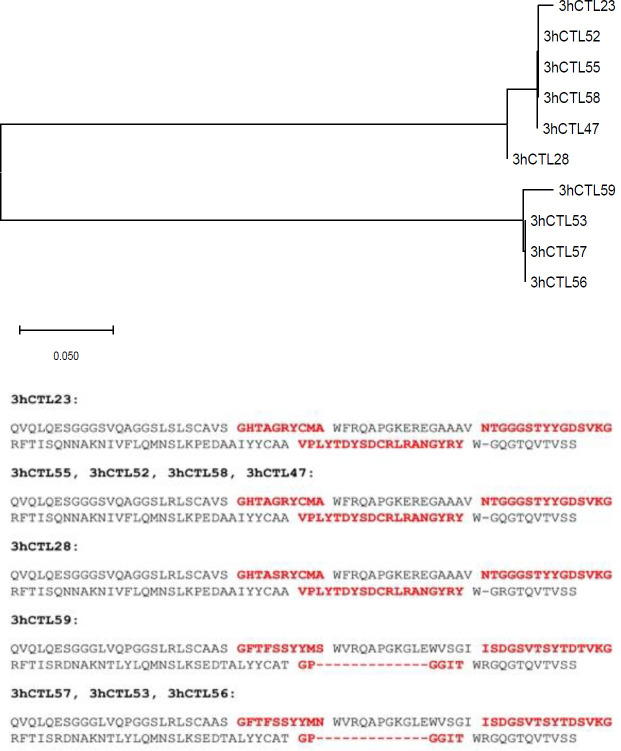
VHH sequencing result. VHH gene of the ten positive colonies was sequenced. Results were analyzed using MEGA-X software (megasoftware.net) which shows the diversity of of two colonies. Amino Acid sequences of nanobodies were shown. CDR1, CDR2, and CDR3 are bolded,in order. 3hCTL55, was selected for further investigations

Polyclonal phage ELISA showed that phage enrichment against hCTLA-4 tended to increase over three consecutive bio-panning rounds (Figure 4). Screening of 64 colonies was done with the PE-ELISA technique to find the specific and strong Nbs against the hCTLA-4 antigen. Comparing positive and negative wells, 29 colonies with at least two-fold absorbance at 450 nm were chosen as positive colonies for further investigation (Figure 5). Results of the sequencing revealed the existence of two main diversities in the library (Figure 6A). Clone No. 55, expressing Nanobody with a longer CDR3 fragment (Figure 6B), was selected for further evaluation.

***VHH expression and purification***

**Figure 7:**
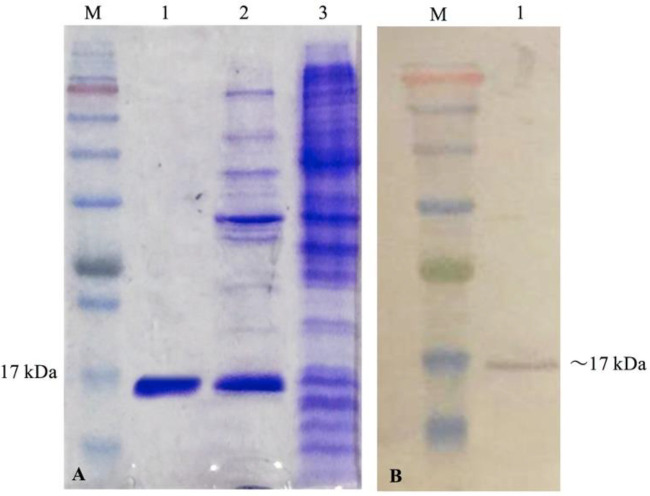
Evaluation of Nanobody expression. *Escherichia coli* WK6 strain containing pHEN6C vector and expressing Nb 3hCTL55 were induced by 1 M IPTG. After periplasmic extraction with osmotic shock, the quality of the purified and concentrated protein was evaluated with %15 SDS-PAGE (A) and western blotting (B). (A) 1; Purified and concentrated protein 2; periplasmic extraction sample after inducing with IPTG 3; Before inducing with IPTG M; protein marker, (B) 1; a single band of Nb M; protein marker

To characterize the selected Nanobody, Nb 3hCTL55, we sub-cloned the VHH gene into the pHEN6C vector and transformed it into an *E. coli WK6* host. IPTG induction resulted in producing Nb in periplasmic space which was released by osmotic shock. Purification and concentration of Nb yielded a sharp band with approximately 17 kDa molecular weight on 15% SDS-PAGE gel (Figure 7A) and nitrocellulose membrane (Figure 7B). 

***Assay***

Antigen specificity

**Figure 8:**
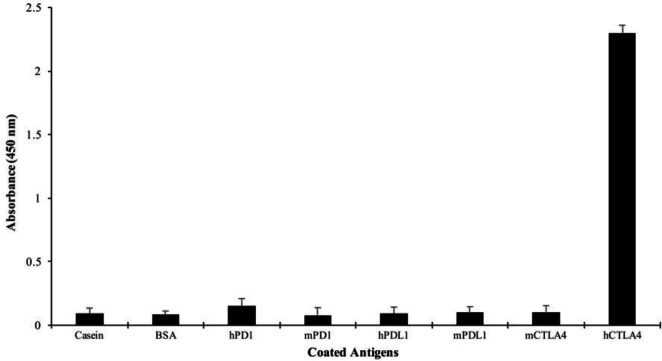
Antigen specificity of Nanobody. The ELISA technique was carried out to assess the specificity of the produced Nanobody 3hCTL55 against human CTLA-4 antigen in comparison with 7 other antigens; casein, BSA, hPD1, mPD1, hPDL1, mPDL1, mCTLA-4

The antigen specificity of Nanobody 3hCTL55 was assessed against eight different proteins with the ELISA technique. Colorimetric readings were done at a wavelength of 450 nm (OD_450_). Results showed Nb 3hCTL55 was not able to detect seven other proteins except hCTLA-4. Nb 3hCTL55 specifically bound hCTLA4 protein and had the maximum absorption of 2.3 which was less than 0.5 for other antigens (Figure 8). 

*Affinity*

**Figure 9:**
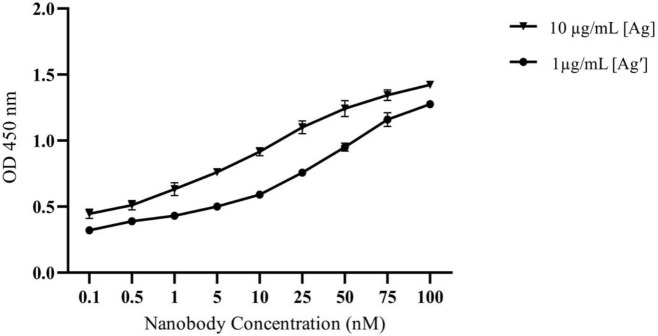
Affinity graph. The affinity of 3hCTL55 Nb binding to hCTLA-4 antigen was calculated by using modified Beatty’s equation

The affinity of 3hCTL55 Nb binding to hCTLA-4 antigen was calculated by using modified equation of Beatty; k_aff =_ n − 1/2 (n [Nb] − [Nb′]), where n is [Ag] / [Ag′], [Ag]; the concentration of 10 µg/mL antigen, [Ag′]; the concentration of 1 µg/ml antigen and [Nb] and [Nb′] are the concentrations of Nanobody at %50 OD_450_ (Max) in the Ag and Ag′ curves. Following the equation, K_aff_ was calculated as 50×10 ^-9^ M. GraphPad Prism 7 (GraphPad Software, Inc., La Jolla, CA, USA), (Figure 9)(21).

*Western blotting on whole cell lysate*

**Figure 10:**
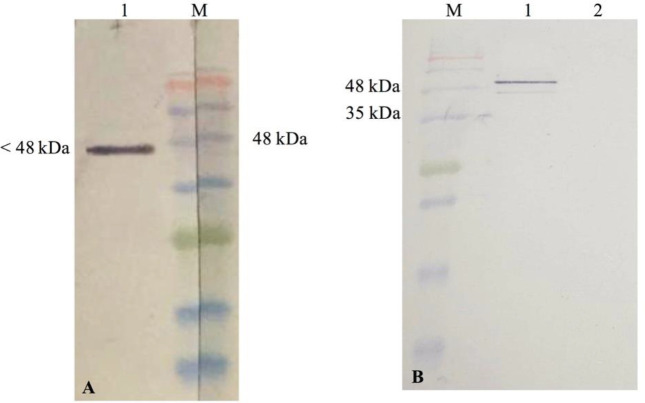
Evaluating the binding ability of Nb 3hCTL55 to CTLA-4 antigen with western blot (A) Applying Nb 3hCTL55 to commercial human CTLA-4 protein. 1; Nanobody-commercial hCTLA-4 antigen interaction M; protein marker, (B) Applying Nb 3hCTL55 to Jurkat and HEK-293 cell lysate as a positive and negative cell, respectively. 1; Jurkat cell lysate and Nb 3hCTL55 2; HEK-293 cell lysate and Nb 3hCTL55 M; Protein marker

We estimated the binding ability of Nb 3hCTL55 to commercial human CTLA-4 protein and also CTLA-4 receptors of the whole-cell lysate of Jurkat cells with the western blot technique. Protein-protein interactions were detected by color development with DAB. As a result, Nbs were able to bind commercial hCTLA-4 protein with a molecular weight of 48 kDa (Figure 10A). Applying Nb 3hCTL55 to the cell lysate in western blotting also showed Nb 3hCTL55 were able to recognize CTLA-4 protein conformation in cell lysate and bind it (Figure 10B). A band with about 50 kDa molecular weight on the nitrocellulose membrane relates to the dimerization of CTLA-4 protein visualized in SDS-PAGE gel.

*Flowcytometry*

**Figure 11:**
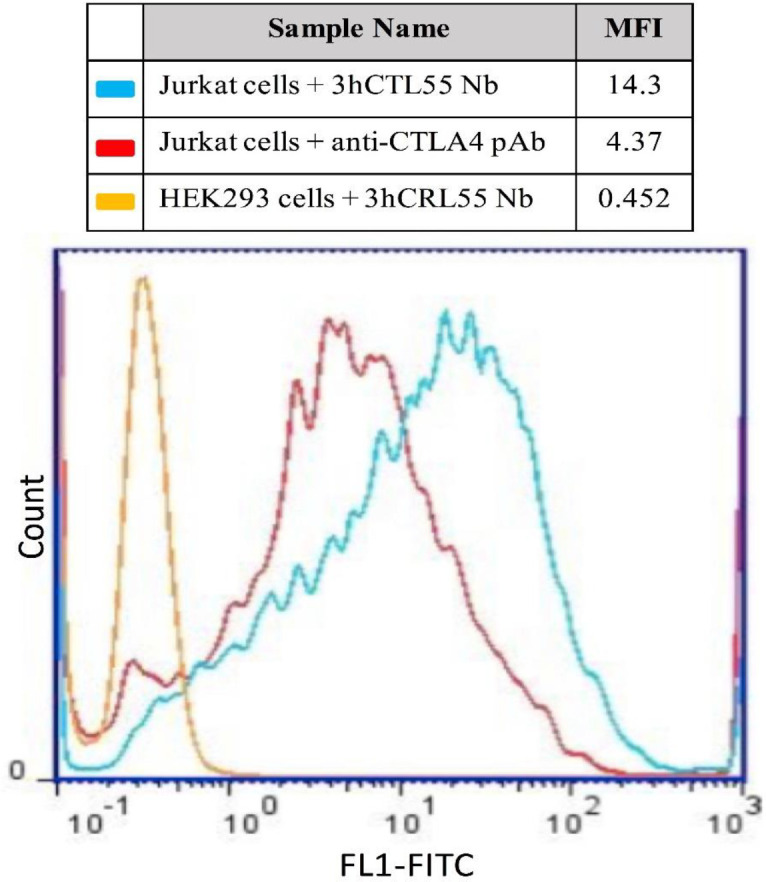
Flowcytometry. Specific interaction between Nb 3hCTL55 and CTLA-4 receptors on cells was evaluated with the Flow-cytometry technique by using the FL1 channel (X-axis). Nb 3hCTL55 was able to specifically bind to the native form of CTLA-4 receptors on Jurkat cells (presenting cell line) and make no interaction with HEK-293 cells. MFI: Median Fluorescence Intensity

To evaluate the specific interaction of Nb 3hCTL55 with CTLA-4 presented on the cell, we performed Flowcytometry. Results confirmed that Nb 3hCTL55 were able to specifically bind to CTLA-4 receptors on Jurkat cells and had no cross-reaction with negative cells, HEK293 (Figure 11). 

## Discussion

Cancer therapy with immune checkpoint inhibitors is a progressive treatment strategy for many cancers. This method relies on blocking immune inhibitory molecules and modulating anti-tumor immune responses (22). CTLA-4, PD1, and its ligand (PDL1) are the principal human inhibitory immune checkpoints that have been overexpressed in a broad range of cancers and are the main target molecules for CPI cancer therapy (23, 24). In 2011, FDA approved the first anti-CTLA-4 monoclonal antibody, (called Ipilimumab (YERVOY®, IgG1)) for advanced melanoma (25). Afterward, because of the positive clinical achievements, several monoclonal antibodies were added to a list of FDA CPI cancer therapy agents. Nivolumab and pembrolizumab (anti–PD-1), atezolizumab, avelumab, and durvalumab (anti–PD-L1) antibodies are some examples. Some other mAbs such as Tremelimumab (anti-CTLA-4, IgG2) are progressing in preclinical and clinical trials to use single or combine with other agents for different types of cancers (12). During the past decades, a great number of small therapeutic proteins with the conventional-monoclonal-antibody-derived regions including Fab, Fc-fusion protein, and scFv, have been developed. Despite many advantages and significant clinical and research applications of classical antibodies, they have some restrictions (26, 27). They are big (150kDa) and also require having both heavy and light variable domains (VH & VL) to bind to antigens. In line with this fact, developing new anti-CTLA-4 molecules with more specific diagnostic and therapeutic potential and fewer limitations are a concern of many pieces of research (28, 29).

Recently, reports show interest in using the smallest antibody fragment of heavy chain antibodies, called VHH or Nanobody, is growing (15, 30). Various renowned biopharmaceutical companies have been concentrating their efforts on these small molecules (31). Significantly, the number of Nanobody drugs entering clinical-trial studies is increasing (29). These nano-antibodies are multi-potent “Lego-like” molecules that can be designed for different purposes. Many Nb-associated agents were developed by manipulating Nb fragments to bind to Nb/Nbs (bivalent/multivalent Nb agent), florescent dye, peptide or protein, radionuclide, drug or toxin, enzyme, nanoparticle, and others (16). 

In the current study, we developed a monoclonal single variable domain antibody against human CTLA-4 antigen. This was followed by gene expression and protein characterization of the selected Nb. There are four well-known human antibody sources; immune library, naïve library, semi-synthetic, and synthetic library. All libraries have several advantages and disadvantages. Low-cost and short-time processing, the possibility to use different forms of antigens, natural antibody maturation, and producing high-affinity antibodies are some advantages of immune libraries (32). In addition, using pure recombinant protein antigens to stimulate the immune system will result in more specific antibody maturation, comparing with using other kinds of antigens such as cell lysate, whole proteins, or pathogen (e.g., toxin) and a mixture of cell lysate and protein (32). Indeed, to achieve a wide variety of benefits in a small library, we boosted a *C. dromedarius* immune system against in-house made recombinant human CTLA-4 protein (hCTLA-4), as previously explained (17). Then, an immune library of VHH-cDNA with a diversity of anti-CTLA-4 nano-antibody repertoires was constructed by using camel PBMC which resulted in 10^8^ binders. 

There are two main display techniques; cell-based display and cell-free display. Phage display is a cell-based technique that brings great benefits as the most common and standard selection technique (33). Therefore, CTLA-4 specific nanobodies from the VHH library, displayed on VCM13 phages, were enriched by three sequential bio-panning rounds (R1, R2, and R3) against commercial CTLA-4 antigen. Performing the phage-ELISA method showed a gradual threefold increase of enrichment factor from Round 1 to Round 3 and confirmed library enrichment. We applied PE-ELISA for monoclonal screening which was followed by nucleotide sequencing of selected colonies. Results revealed the existence of diversity in two clones in the library.

Structurally, Nanobody consists of four conserved framework regions (FW1/2/3/4) and three hyper-variable antigen-binding loops, called complementarity determining regions (CDR1/2/3). The CDR3 loop of nanobodies has a finger-like structure with a mean of 18 amino acid residues that enable nanobodies to bind antigens. The longer the CDR3 sequence, the better interaction with antigen (29). For this reason, we chose one Nanobody of the cluster with a longer CDR3, with 19 amino acid residues (Figure 6) named 3hCTL55. Followed by the large-scale expression of Nb 3hCTL55, more characterization was done after periplasmic extraction and Ni-affinity chromatography purification and concentration. 

The specificity of Nb 3hCTL55 to hCTLA4 antigen was assessed by the ELISA technique. Compared with seven other antigens, Nb 3hCTL55 specifically detected immobilized commercial hCTLA-4 protein and had no cross-reaction with other proteins. Additionally, we confirmed the specific binding of Nb 3hCTL55 to both commercial CTLA-4 antigens and CTLA-4 receptors from CTLA-4 expressing cell lysates with western blotting.

The ability to recognize the native form of an antigen is a particular feature of an antibody to make the antibody-antigen interaction. This feature exposes the specificity and affinity of an antibody to an antigen (34, 35). Using the Flowcytometry technique by Wen *et al*. revealed the developed anti-peptide PD1^125-126^ Nanobody was able to bind PD1 antigen and block the binding site of the PD1/PDL1 interaction interface (36). We applied Flowcytometry which showed Nb 3hCTL55 were able to detect CTLA-4 antigens on the surface of the CTLA-4 expressing cells. We compared flow cytometry peaks relating to the interaction of anti-CTLA4 polyclonal antibody and Nb 3hCTL55 with native CTLA-4 antigens on the cell surface. Results explained Nb 3hCTL55 was able to properly detect native CTLA-4 antigens and bind them. By using the modified Beatty method, we calculated the Nb 3hCTL55 affinity to CTLA-4 antigen as 50×10^-9^ M. Banihashemi *et al*. showed using a cocktail of two developed nanobodies was able to shift the Flow-cytometry binding peak to the right side. This can be the result of the synergy effects of using two nanobodies (37). 

Blocking human immune checkpoint by using Nbs is now under investigation. In 2018, Wan *et al*. examined the effectiveness of in-house produced recombinant anti-CTLA-4 Nb16 molecules on melanoma-bearing mice. Results showed Nb16 delayed tumor growth and increased survival time (38). 

Later in 2019, Wen *et al*. used a humanized Nanobody phage display library to screen and select a specific Nanobody against PD1 molecules. They showed selected PD1-Nb-B20 nanobodies potentially bound to the PD1 molecules and blocked the interaction interface of PD1/PDL1 on the surface of over-expressing PDL1 cells (36). Recently, in 2020, researchers constructed a camelid immune library against PDL1 molecules, showed three anti-PDL1 nanobodies specifically bound to PDL1 molecules confirmed to have a high affinity to PDL1(39).

In this article, we represented a novel and unique anti-CTLA-4 Nanobody (3hCTL55) with a particular sequence and specifications that were totally different from what was presented in the Wan *et al*. article. We examined the specificity of our Nanobody which was unique and showed a noticeable affinity for detecting and binding to CTLA-4 proteins on the surface of the cells.

## Conclusion

We developed a novel anti-CTLA-4 Nanobody, 3hCTL55, selected from a high-quality immune library by phage display technique that was capable of specifically binding to CTLA-4 antigen in native form. It can be effective for further study of cancer diagnosis and cancer therapy purposes.

## Authors’ Contributions

NS, MB Study conception or design; ZN, FK Data analyzing and draft manuscript preparation; MB, MH Critical revision of the paper; NS, MB Supervision of the research; MB Data Processing, Collection, Perform Experiment.

## Conflicts of Interest

No competing financial interests exist. 
